# Evaluating the significance of *Toxoplasma gondii* sporozoite antibodies in cats: a pilot study

**DOI:** 10.1186/s13071-024-06553-6

**Published:** 2024-12-02

**Authors:** Janelle Scott, Arianne Morris, Jennifer Hawley, Andrea Valeria Scorza, Michala Henriksen, Michael Lappin

**Affiliations:** 1 College of Veterinary Medicine and Biomedical Sciences, Colorado State University, Fort Collins, CO USA; 2 College of Veterinary Medicine, North Carolina State University, Raleigh, NC USA

**Keywords:** *Toxoplasma gondii*, Sporozoite, Oocyst, Cat

## Abstract

**Background:**

People can acquire *Toxoplasma gondii* infection by ingestion of sporulated oocysts passed in cat feces; whether this route is common in cats is unknown. The primary objectives of this study were to (a) adapt a commercially available enzyme-linked immunosorbent assay (ELISA) for the detection of *T. gondii* tachyzoite IgG antibodies in feline sera to detect *T. gondii* sporozoite IgG antibodies, (b) utilize the ELISA to confirm that exposed cats can mount an antibody response to sporozoites, (c) estimate the prevalence of sporozoite antibodies in naturally exposed cats, and (d) evaluate associations between the serologic status of naturally exposed cats and clinical signs that could be caused by toxoplasmosis.

**Methods:**

To generate positive control sera, three male cats were orally inoculated with approximately 100,000 sporulated oocysts of the ME49 strain of *T. gondii*. A human antisporozoite antibody ELISA was then adapted for use with cat sera. Detectable levels of antisporozoite IgG were found in two of the three experimentally inoculated cats. The sera of 100 healthy cats and 295 clinically ill cats were assessed in the prototype sporozoite ELISA and a commercially available tachyzoite ELISA.

**Results:**

The ELISA estimated that prevalence of antisporozoite IgG was 2% in healthy cats and 3.1% in clinically ill cats; in contrast, the overall estimated prevalence of antitachyzoite IgG was 15%. Only two of 395 cats (0.5%) had both antisporozoite and antitachyzoite IgG.

**Conclusions:**

While experimentally infected and naturally exposed cats developed antisporozoite antibodies, the low prevalence did not allow for the evaluation of associations among clinical signs.

**Graphical Abstract:**

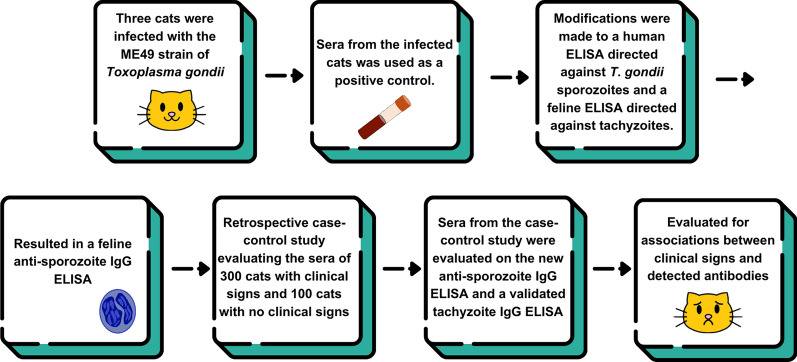

## Background

*Toxoplasma gondii* is a global intracellular protozoan capable of infecting mammals and birds. The seroprevalence of *T. gondii* varies greatly worldwide [1], but in many regions it is still a health and economic concern for humans, wildlife, and domestic animals. Most infected cats do not exhibit clinical signs; however, immunosuppressed cats can exhibit fever, diarrhea, neurologic abnormalities, respiratory distress, uveitis, or chorioretinitis [2]. Clinical disease is similar in intermediate hosts (non-felids). Most will have no to only mild clinical signs (myalgia, mild lymphadenopathy). Immunosuppressed individuals and fetuses are the most severely affected; clinical signs include spontaneous abortions, congenital deformities, retinochoroiditis, neurologic deficits, seizures, or pneumonia [3].

Cats are the definitive host; the sexual phase of the *T. gondii* life cycle that results in oocyst shedding only occurs in felids. The complex *T. gondii* life cycle has been previously described [4]. In short, there are three different primary *T. gondii* life stages: (1) sporozoites that develop within fecal oocysts, (2) tachyzoites that rapidly divide and spread to various organ systems, and (3) bradyzoites housed within tissue cysts. Exposure to *T. gondii* usually occurs via ingestion of either sporulated oocysts from the environment or tachyzoites or bradyzoites in the tissue of intermediate hosts. Vertical transmission is also possible as transplacental transmission can play an important role in people and cats. Transmammary exposure has also been documented in cats and some intermediate hosts [5].

Ingestion of any of the three life stages can result in *T. gondii* infection. Historically, determining the specific route of horizontal transmission (ingestion of bradyzoites or tachyzoites versus ingestion of sporozoites) had to be inferred from the host’s risk factors. The route of infection in humans could not be objectively evaluated until a *T. gondii* sporozoite-specific antigen was identified and expressed [6]. Using serology to evaluate the route of exposure has emphasized that oral ingestion of sporozoites can play a role in human infection with *T. gondii*. In one study, 43% of the serum samples from pregnant Chilean women had IgG antibodies to a sporozoite-specific protein [7].

In cats, most of the previous research on *T. gondii* transmission was directed toward the consumption of encysted bradyzoites in meat as this was thought to be both the most common [8] and most efficient route [9, 10]. Given the carnivorous nature of felines, less focus has been placed on the ingestion of *T. gondii* sporozoites. Cats frequently groom and it has been proposed that exposure could occur via ingestion of sporulated oocysts contaminating skin or hair or by drinking contaminated water. As there was not a serologic method to determine *T. gondii* sporozoite antibodies in cats, exposure to this life stage via ingestion may have been historically underestimated. It has also been proposed that the route of exposure and life stage of *T. gondii* could impact whether clinical disease occurs [9]. Therefore, understanding the route of exposure has potential implications for disease management and preventative measures.

Based on necropsy, histopathology, and the observation of unsporulated oocysts in the feces of infected cats, it is commonly accepted that the sporulation of oocysts occurs only in the environment after infected cats shed the oocysts in their feces [4, 11]. If oocyst sporulation occurs only after the oocysts are passed in feces, the presence of antisporozoite antibodies should indicate that the infection occurred via the ingestion of sporulated oocysts from the environment. Therefore, the primary purpose of this study was to (a) adapt a commercially available enzyme-linked immunosorbent assay (ELISA) for the detection of *T. gondii* tachyzoite IgG antibodies in feline sera to detect *T. gondii* sporozoite IgG antibodies, (b) utilize the ELISA to confirm that exposed cats can mount an antibody response to sporozoites, (c) estimate the prevalence of sporozoite antibodies in naturally exposed cats, and (d) evaluate associations between the serologic status of naturally exposed cats and clinical signs that could be caused by toxoplasmosis.

## Methods

### Generating positive control sera

Before designing a prototype ELISA targeting antibodies to *T. gondii* sporozoites, it was necessary to generate positive control sera. The project was approved by the Institutional Animal Care and Use Committee (protocol 17-7341A) and three male cats were transferred from a research facility to Colorado State University. On day 0, the three cats were shown to be seronegative for *T. gondii* antibodies on a commercially available feline ELISA directed against tachyzoites [12] [https://vetmedbiosci.colostate.edu/vdl/]. Each of the three cats were manually restrained and orally inoculated with approximately 100,000 sporulated oocysts of the *T. gondii* ME49 strain (courtesy of Dr. JP Dubey, US Department of Agriculture). The cats were group housed during the day and enclosed in separate cages at night to enable collection of feces for microscopic examination of *T. gondii* oocysts. When possible, feces were collected twice daily. One gram of feces was mixed with Sheather’s sugar solution (specific gravity 1.26), centrifuged at 800*g* for 10 min and then examined under the microscope using 10× magnification. The pupils of the cats were dilated for fundic examinations to assess for lesions consistent with toxoplasmosis on day 22 postinfection.

Previously developed commercially available *T. gondii* IgM and IgG ELISAs, which use a tachyzoite lysate as the antigen, were used to analyze sera from the cats on day 0, 3, 7, 10, 14, 17, 21, 28, 35, 42, 49, and 56 [12] [https://vetmedbiosci.colostate.edu/vdl/]. All three cats had detectable levels of antitachyzoite IgM and IgG by day 28. The sera were then stored at –80 °C.

### Development of the prototype sporozoite ELISA using feline sera

To create an ELISA capable of detecting IgG directed against *T. gondii* sporozoite-specific antigens, conditions used in the human ELISA directed against sporozoites [6] and the feline ELISA directed against tachyzoites [12] were evaluated to develop a prototype assay. Sera from days 49 and 56 after sporozoite inoculation were chosen as the likely positive control in the initial titrations. For the negative control, sera of specific pathogen-free cats from previous studies (stored at –80 °C until assayed) were used.

Multiple microELISA plates and binding buffers were assessed. A checkerboard approach was used to optimize the sporozoite antigen and secondary antibody concentrations. In the finalized prototype assay, the previously characterized [6]. *T. gondii* sporozoite antigen (*T. gondii* embryogenesis-related protein; courtesy of Dolores Hill, USDA) was diluted to 1 μg/ml in 50 mM carbonate/bicarbonate buffer (pH 9.6). This was then used to coat 96-well microtiter plates (Corning Incorporated Kennebunk, Maine) with 100 μl per well for 60 min at 37 °C. One row of the plate was left without antigen as a physical buffer. The antigen-coated wells were washed three times with TBS-MT (50 mM Tris at pH 7.4, 150 mM NaCl, 5% non-fat milk powder, and 1% Triton X-100). After each wash, the plates were blotted dry on paper towels. The feline sera were diluted with phosphate-buffered saline with Tween 20 (PBS-Tween 20) to a concentration of 1:64, and 100 μl of each diluted serum was added to each well; all controls and samples were run at minimum in triplicate wells. The plate was then allowed to incubate at room temperature for 1 h. Washing of the antigen-coated wells was repeated as previously described. Goat anti-cat IgG–HRP conjugate (Seracare, Milford, Massachusetts) was used as the secondary antibody and was diluted 1:1500 in TBS–milk (50 mM Tris at pH 7.4, 150 mM NaCl, 5% non-fat milk powder); then 100 μl was added to each antigen-coated well. Again, the plate was washed as previously described. The antigen-coated wells were then rinsed once with 200 μl distilled water. A final incubation of 20 min at room temperature was performed after adding 100 μl of chromagen TMB substrate (Seracare, Milford, Massachusetts) to the entire plate. One hundred microliters of 8N H_2_SO_4_ was added as a stop solution to each well at the end of the incubation. The optical density of each well was then determined in a microplate reader with 450 nm filter (Thermofisher Scientific, Watham, Massachusetts).

The finalized prototype sporozoite ELISA was then performed on banked sera of 25 other research cats. Two outliers were removed due to their extremely high absorbance values; these were suspected to be related to prior exposure. The mean absorbance (0.025) and standard deviation (0.009) was then calculated for the 23 samples. A cut-off point of the mean plus two standard deviations was then chosen (0.043). To determine a multiplication factor to be used across plates to help correct for any interassay variation, the mean plus two standard deviations was divided by the negative control absorbance level (0.052/0.043 = 1.2). The positive cut-off point for each plate was then determined by taking the multiplication factor of 1.2 times the mean of the negative controls on that plate. Absorbances above this cut-off point were considered IgG positive for antibodies against *T. gondii* sporozoites. Temporally collected serum samples from the three cats that were orally inoculated with sporulated oocysts were then assayed via both the IgG tachyzoite ELISA and the IgG sporozoite ELISA.

The interassay variation of the IgG sporozoite ELISA was determined by measuring the mean absorbance values of the negative and positive controls on six plates. A ratio of the mean positive control to the mean negative control was also calculated for each plate. The standard deviation of the mean negative control wells and the mean positive control wells were calculated across plates. The coefficient of variation was then determined by dividing the standard deviation by the mean × 100 for both the positive and negative controls.

### Using serology to investigate the possibility of sporulation within the gastrointestinal tract

Stored sera from cats (*n* = 5) orally inoculated with Mozart *T. gondii* bradyzoites were available for study [13]. Sera from day 0, 14, 28, and 56 were thawed and then assayed in both the commercially available IgG tachyzoite ELISA and the prototype IgG sporozoite ELISA.

### Retrospective case–control study

To evaluate potential associations between the results of the *T. gondii* antibody assays and clinical signs, a retrospective case–control approach was chosen. For the case arm of the study, banked feline sera previously submitted to the Colorado State University Veterinary Diagnostic Laboratory for the commercially available *T. gondii* antitachyzoite IgM/IgG ELISA were utilized. A convenience sampling and snowball approach was used to choose the sera of 300 cats who met the following criteria: clinical suspicion of infection and at least one of the following clinical signs: fever, ataxia, seizures, anterior or posterior uveitis, dyspnea, icterus, or myocarditis. The sera were submitted by practicing veterinarians; therefore, these cats represent naturally acquired infections.

For the control group, a convenience sampling of previously banked sera from 100 healthy blood donor cats was utilized. These cats were negative for FeLV and *Dirofilaria immitis* antigen, FIV antibody, and DNA of the feline *Hemoplasma* spp., *Bartonella* spp., and *Ehrlichia* spp.

Next, all 400 sera were thawed and assayed in both the commercially available antitachzyoite IgG ELISA and the prototype antisporozoite IgG ELISA. When validating the data, five sera samples were excluded due to missing data or transcription errors. Therefore, the ELISA results of 295 of the clinically ill cats and 100 of the healthy cats remained for analysis.

### Statistical analysis

The clinical signs of the cats found on the submission forms were divided into four categories: fever, ocular, neurologic, or respiratory. The serological test results were then stratified by groups and presented descriptively. To assess associations between clinical signs and serology status, Fisher’s exact test was performed utilizing the online open source software OpenEpi [14]. Significance was defined as *p* < 0.05.

## Results

### Cats inoculated orally with sporozoites to generate positive sera

All three cats fed sporulated oocysts remained healthy and no clinical signs were observed over the course of the study. On day 22, one cat had a normal fundic examination. One cat had three focal hypopigmented lesions in the nontapetal area, unilaterally, and one cat had a focal hyperpigmented lesion bilaterally.

Each of the cats developed detectable levels of IgG to tachyzoites on the commercially available ELISA. Antibodies were first detected on day 17 (cat 1) and 28 (cat 2) and were still present on the last sample on day 56. On the prototype ELISA, IgG to sporozoites was detected in sera from two of the three cats by 56 days postinoculation. Only one cat was found to be shedding oocysts during the study (day 46) and this cat developed IgG to sporozoites.

### Finalized prototype sporozoite IgG ELISA

The interassay variation for the sporozoite ELISA was 19% for the negative controls and 14% for the positive controls.

### Serology results of cats exposed via bradyzoites

All of the cats orally inoculated with bradyzoites had detectable levels of IgG directed against *T. gondii* tachyzoites by day 56. However, none of these cats had detectable levels of IgG directed against *T. gondii* sporozoites.

### Serology results of retrospective case–control study

In this pilot study, anti*T. gondii* sporozoite IgG antibodies were detected in 11 of the 395 cats (Table 1). In cats with clinical signs in this study, the estimated *T. gondii* sporozoite antibody prevalence rate was 3.1% (Table 1). Comparatively, in apparently healthy cats in this study, the estimated sporozoite antibody prevalence rate was 2% (Table 1).

**Table 1:** Contingency table of *Toxoplasma gondii* IgG ELISA results, using sera from naturally exposed cats, in both the prototype sporozoite and the commercial tachyzoite assay

	Tachyzoite positive	Tachyzoite negative	Total
Clinically ill cats			
Sporozoite positive	2	7	9 (3.1%)
Sporozoite negative	57	229	286 (96.9%)
Total	59 (20%)	236 (80%)	295
Healthy cats			
Sporozoite positive	0	2	2 (2%)
Sporozoite negative	3	95	98 (98%)
Total	3 (3%)	97 (97%)	100

### Analysis of sporozoite serology status with regard to clinical signs

Using the serology results of the cats with clinical signs, contingency tables were created using the clinical sign category (fever, ocular, neurologic, or respiratory) as a factor and the sporozoite serology status for disease status. The seven clinically ill cats with sporozoite IgG antibodies were distributed as follows: fever = 1, ocular = 2, neurologic = 2, and respiratory = 2. Statistically significant associations between the category of clinical signs and the sporozoite serology status were not found (Fisher’s exact test, *p* = 0.5669, odds ratio [OR] = 1.147, confidence interval [CI] 0.1334–6.245). There were only two clinically ill cats who had both sporozoite and tachyzoite IgG antibodies; one cat manifested neurologic signs while the other cat showed ocular clinical signs.

## Discussion

To the authors’ knowledge, this is the first report using a prototype ELISA to detect *T. gondii* antisporozoite antibodies in cats. Evidence of antisporozoite antibodies were found in multiple naturally exposed cats. However, given the low overall prevalence of antisporozoite IgG in this study, this route of infection is likely less common in the domestic cat population. This is in contrast to a previous study supporting that infection via sporozoite ingestion can be common in humans [6].

Previous studies support the concept that oral ingestion of sporozoites is also a less successful route of infection in cats compared with bradyzoites [9]. We suspect this is why one experimentally inoculated cat described here did not develop detectable levels of IgG on the sporozoite ELISA by day 56. In addition, only one of the three experimentally inoculated cats was found to be shedding oocysts; this cat developed IgG directed against both sporozoites and tachyzoites. The low detection of oocysts by fecal flotation in our study is not unexpected. Cats shed *T. gondii* oocysts for a relatively short time and detection of oocysts from feline feces is rare [13]. The sensitivity of microscopic examination for detection of *T. gondii* oocysts is low; *T. gondii* oocysts were detected by microscopic examination in 2% or less of the cats in two studies performed in the USA [15, 16]. Further, a study reported a PCR assay that is more sensitive than microscopic examination of feces and it is at least as sensitive and specific as the bioassay [17]. It is possible that low levels of oocysts were missed on flotation in our study. For both experimentally inoculated cats with fundic lesions, the lesions appeared chronic and were not suspected to be secondary to toxoplasmosis.

Our study results further support the concept that sporulation of *T. gondii* oocysts only occurs after the oocysts are excreted in feline feces. The sexual phase of the *T. gondii* life cycle culminates in the production of oocysts containing a sporoblast. Two sporocysts, containing four sporozoites apiece, develop when the oocyst is exposed to oxygen [9]; therefore, sporozoites should not develop until passed in the cat feces.

In contrast to *T. gondii*, there are protozoa such as *Cryptosporidum felis* [18] where sporulation does occur in the feline gastrointestinal tract. If this in fact occurred with *T. gondii*, then a positive sporozoite antibody assay would not be specific for the ingestion of sporulated oocysts. The cats orally inoculated with Mozart bradyzoites did not develop detectable levels of IgG against sporozoites. Given this finding and the current understanding of the *T. gondii* life cycle, it strongly suggests that when sporozoite IgG antibodies are detected in feline sera the cat was exposed to *T. gondii* via ingestion of sporulated oocysts. Exposure to sporulated oocysts could possibly occur from self-grooming after being exposed to contaminated soil, ingestion of contaminated water, or mutual grooming of an infected housemate.

In our retrospective case–control study, from the 295 clinically ill cats and 100 healthy cats, only 11 cats had detectable levels of *T. gondii* sporozoite IgG (Table 1). In apparently healthy cats and clinically ill cats, the estimated prevalence rates for sporozoite IgG were 2% and 3.1%, respectively. The method we used to set our cut-off points erred on the side of increased sensitivity and so negative results should be uncommon. However, there is a concern for precision across the plates. Given the 14% and 19% coefficients of variation for positive and negative controls, respectively, there could be false positives or false negatives for absorbance levels that were near the cut-off point.

A previous study evaluating the seroprevalence of *T. gondii* in cats in the USA found 28.7% of clinically ill cats were positive for IgG only or IgG and IgM on a tachyzoite ELISA [12]. Our study found a similar seroprevalence: 20% of clinically ill cats had IgG directed against tachyzoites. Only two of the 395 cats, both who were clinically ill, were seropositive on both the sporozoite and tachyzoite ELISAs. In contrast to the tachyzoite seroprevalence, our finding of a low prevalence rate of cats with sporozoite is not unexpected. It further supports the idea that most cats are exposed to *T. gondii* via ingestion of bradyzoites or tachyzoites. As this is the first study using serology to evaluate sporozoite-induced infections in cats, it is not known how long antibodies remain detectable. It is possible that some of the cats we evaluated had previously ingested *T. gondii* sporozoites and (a) they never mounted a detectable response or (b) they mounted a response, but the antibody levels were below the level of detection.

The route of transmission seems to play a role in both the infectivity and pathogenicity of *T. gondii* [9]. Additionally, there is some evidence that it could play a role in the clinical signs observed [19]. Ashour et al. [19] found that mice orally infected with *T. gondii* were more likely to have necrotic damage to ocular tissue, while mice who were congenitally infected were more likely to have proliferative ophthalmic lesions. In our study, no statistically significant associations between sporozoite IgG levels and the type of clinical signs were found. However, this may be related to study design. The sera chosen for the retrospective case control study was readily available through the Center for Companion Animal Studies, which is a section of the Colorado State University Veterinary Diagnostic Laboratory. A convenience sampling was chosen, utilizing samples submitted from practitioners with a clinical suspicion of toxoplasmosis. Thus, meeting of the case definition was reliant on secondary information on the submission forms accompanying the samples. Potential limitations include accuracy of these forms and the very limited information regarding each individual case. Furthermore, we were hindered by the lack of knowledge of the seroprevalence of antibodies to *T. gondii* sporozoite antigen when the study was designed. Using the estimated prevalence of *T. gondii* sporozoite IgG found in this study, the appropriate sample size needed to accurately evaluate for disease associations could be utilized in future studies.

An ideal next step would be to perform a western blot and Coomassie dye stain to evaluate that the detected feline IgG antibodies were specifically binding to the *T. gondii* embryogenesis-related protein. We were unable to obtain more *T. gondii* embryogenesis-related protein to proceed. However, given the extensive mapping and testing of this protein in the primary study [6], we believe it is likely that the prototype ELISA is detecting feline antibodies binding with the sporozoite protein of interest. However, our study is limited by the inability to fully confirm this finding as this antigen is no longer available. Further studies should be performed to corroborate the results described here and could use recombinant proteins from sporozoites/oocysts like CCp5A and OWPI [20].

## Conclusions

In this study, two of the three cats orally inoculated with *T. gondii* sporulated oocysts had detectable levels of IgG on the prototype sporozoite ELISA. A group of five cats that were infected with *T. gondii* Mozart bradyzoites were negative on the prototype *T. gondii* sporozoite antibody assay; this provides further confirmation that *T. gondii* sporulation only occurs after oocysts are passed in feces. We also found a low estimated prevalence of antisporozoite IgG in cats exposed to *T. gondii* in the field; this further supports the idea that most cats are exposed to *T. gondii* via ingestion of bradyzoites or tachyzoites. To our knowledge, this is the only study estimating the prevalence of sporozoite antibodies in naturally exposed cats.

## Data Availability

No datasets were generated or analysed during the current study.

## Abbreviations

ELISA: Enzyme-linked immunosorbent assay
USDA: US Department of Agriculture
TBS-MT: Tris-buffered saline with Tween-20
PBS-Tween 20: Phosphate-buffered saline with Tween-20
HRP: Horseradish peroxidase
FeLV: Feline leukemia virus
FIV: Feline immunodeficiency virus
DNA: Deoxyribonucleic acid
